# The role of learning and training in supporting emotional resilience in security forces

**DOI:** 10.3389/fpsyg.2026.1854418

**Published:** 2026-05-28

**Authors:** Marek Sedlacík, Radovan Soušek, Michaela Stanková, Jan Cáp

**Affiliations:** 1 Faculty of Military Leadership, University of Defence, Brno, Czechia; 2 Faculty of Transport Engineering, University of Pardubice, Pardubice, Czechia; 3 Faculty of Law and Public Administration, University of Finance and Administration, Prague, Czechia

**Keywords:** education system, employability, performance, security forces, training

## Abstract

Emotional resilience is a key psychological competence enabling individuals to adapt positively to stress, overcome adversity, and maintain wellbeing in both personal and professional contexts. In dynamic, high-stakes environments such as security forces, it plays a critical role in professional performance, decision-making, and mental stability. This study analyses expert feedback from 224 personnel with operational experience to identify educational and training elements that enhance readiness, adaptability, and the capacity to cope with demanding situations. Rather than directly measuring emotional resilience, the research focuses on training-related factors perceived as contributing to its development. Using qualitative content analysis supported by statistical testing, several weaknesses within the current education and training system were identified. Based on the respondents' insights, the study proposes a revised training framework with a stronger emphasis on practical training, teamwork, international cooperation, and the involvement of external experts. The findings highlight the importance of structured educational interventions and continuous training programmes in fostering emotional resilience and strengthening professional competence within security forces.

## 1 Introduction

Working in the security forces is in many respects significantly more demanding than work in other professions. In addition to maintaining a clean criminal record and meeting the required educational standards, applicants must undergo psychological testing as well as physical and medical examinations (see, e.g., Drozd et al., 2025). This is because the work is both mentally and physically demanding: it involves exposure to high levels of stress and often requires rapid decision-making under pressure. Duties frequently include night shifts and work at weekends and on public holidays. In addition, their members are called upon to assist in responding to emergencies and crisis situations as part of the Czech Republic's crisis management system (Tušer et al., 2021). Taken together, these factors can have a considerable negative impact on an individual's wellbeing and emotional resilience.

Over the past several decades, as emotional resilience has moved to the forefront of scholarly interest, a number of conceptual frameworks have emerged, each emphasizing different aspects of the construct. According to

(Farchi and Peled-Avram 2025), resilience research is commonly divided into approaches that conceptualize resilience as a personal trait, those that understand it as a dynamic process, and those that highlight the role of environmental and systemic factors. (Farchi and Peled-Avram 2025) themselves propose an integrative framework that connects these fragmented perspectives through the so-called ART framework (Acknowledgment, Reframe, and Tailoring). In line with this conceptual approach, emotional resilience in the present study is understood not merely as a relatively stable personality characteristic, but as a developable psychological capacity that can be systematically strengthened through targeted educational and training activities. Within the environment of security forces, education becomes a fundamental pillar for building this capacity. Through structured educational programmes, individuals gain a deeper understanding of the psychological mechanisms underlying stress responses, adaptation processes, and coping strategies. Such theoretical grounding provides a cognitive map that helps personnel recognize early signs of strain, interpret their own reactions more accurately, and make informed decisions about how to regulate their emotions in demanding situations.

Training represents the second essential component in this developmental process. While education provides the conceptual framework, training enables personnel to apply this framework in practice. Repeated exposure to realistic, challenging, and crisis-oriented scenarios allows individuals to translate theoretical knowledge into automatic behavioral and emotional responses. In this way, training serves as a bridge between understanding and action by strengthening procedural memory, improving situational awareness, and enhancing the ability to remain functional under pressure. Importantly, such experiences reduce the unpredictability of real-life operations by creating a repertoire of practiced responses accessible even under high-stress conditions.

Together, education and training form a comprehensive system of psychological preparation closely linked to the development of emotional resilience. This integrated approach supports both effective coping with acute stressors and long-term adaptability in the face of cumulative operational demands. Resilience cultivated through these means contributes to individual wellbeing, reliable performance in critical situations, and reduced error rates, while also enhancing teamwork and communication. Education and training thus constitute a core mechanism through which emotional resilience can be intentionally developed and sustainably maintained.

According to Wolke et al. (2025), resilience research primarily relies on two analytical approaches: a variable-centered approach and a person-centered approach. Complementarily, Maltby and Hall (2022) differentiate between direct measurement of resilience using dedicated instruments and indirect operationalization of resilience inferred from resilience-related factors or adaptive outcomes. An empirical example of such an indirect approach is provided by Kaniasty et al. (2025), who demonstrate that subjective confidence in one's ability to cope with future situations – reflecting individuals' perceptions of their own capacity to manage challenges—can serve as an indicator of resilience. In this article, we draw on the conceptual frameworks proposed by Kaniasty et al. (2025) and, instead of directly measuring emotional resilience, we examine factors that are theoretically or empirically associated with resilience. Although traditional approaches to the analysis of emotional resilience have been widely examined (Smaliukiene et al., 2024, 2025), there remains a research gap in understanding how targeted training, understood as a factor allowing for the indirect examination of resilience, may contribute to strengthening resilience capacities in high pressure professions such as policing, firefighting, and the wider security services in the Czech Republic.

This study contributes to the literature by identifying practitioner-informed training factors that may support the development of emotional resilience. Building on the well-established connection between education, training, resilience, and wellbeing, the study adds to the current discussion on readiness in public service professions. The findings can assist policymakers, training designers, and educators in developing more integrative, experiential, and evidence-based programmes that prepare individuals for the psychological demands inherent in service performance. Ultimately, the results may offer a foundation for international collaboration and the exchange of best practices in resilience education across security and emergency sectors.

The remainder of this article is organized as follows. Section 2 reviews the relevant literature on emotional resilience, professional training, and psychological preparedness in public service professions. Section 3 sets out the aim of the study, its motivation, and its contribution, and formulates the research questions and hypotheses. Section 4 describes the data, the questionnaire structure, and the analytical methods employed. Section 5 presents the empirical results, divided into findings related to the research questions (Section 5.1) and those related to the hypotheses (Section 5.2). Section 6 discusses the implications of the findings for training practice and organizational policy within the security forces, outlines the study's limitations, and offers suggestions for future research. Section 7 concludes.

## 2 Literature review

Moreno et al. (2024) conducted an in-depth analysis of research on resilience training programmes. According to their findings, existing studies can be categorized into three groups: mindfulness-based resilience interventions, neurobiology-based resilience interventions, and a final group comprising studies that do not fit into either of the previous two categories. Their detailed review of 32 published studies indicates considerable variation in the structure and design of individual interventions. Nevertheless, most studies reported positive outcomes for the variables examined, particularly clinical and performance indicators such as stress, anxiety, and decision-making.

Moreno et al. (2024) emphasize that future research should focus, among other priorities, on identifying the key professional factors required to develop a robust and effective resilience-focused intervention programme. However, Singh et al. (2024) point out that it is essential to distinguish between individuals and teams in such analyses, as individual resilience is shaped by different factors than group resilience.

In addition to the 32 studies analyzed in Moreno et al. (2024), recent research also includes, for example, Anum et al. (2025). Drawing on questionnaire data from 109 police cadets in Ghana, Anum et al. (2025) examined their stress-coping strategies, emotional intelligence, and resilience in relation to length of service and different levels of education. Their findings indicate an urgent need to address occupational stress and to strengthen mental health support within the Ghana police service. Many of the identified problems arise from specific sociocultural conditions, as the police service remains deeply influenced by a masculine organizational culture that discourages displays of vulnerability and often interprets them as signs of weakness. Although there are signs of progress through increasing gender diversification, cultural reforms remain very limited.

Cao et al. (2023) conducted a psychological assessment of 1,110 soldiers in China during the COVID-19 pandemic, a period that further intensified the already demanding nature of military service. Their findings indicate that maintaining military culture significantly supports soldiers in preserving their mental health. However, it is also essential to strengthen soldiers' contact with family members and friends, who not only help encourage and motivate them but may also serve as individuals capable of reporting requests for assistance or guidance to the relevant authorities on behalf of a soldier in need.

Social support in the form of strong interpersonal relationships has also been shown to be an important factor in reducing symptoms of post-traumatic stress disorder (PTSD). Based on the responses of 113 police officers in the United States, McCanlies et al. (2017) demonstrated a statistically significant negative association between social support, resilience, and components of wellbeing on the one hand and PTSD symptoms on the other.

Bekesiene et al. (2021) also emphasize the importance of communication in their study, which examined the training of behavioral competencies among military commanders and leaders. Drawing on information obtained from 220 soldiers from all units of the Lithuanian armed forces and 37 military commanders and leaders, they identified and ranked 12 key factors, including, for example, a leader's ability to persuade others and the capacity for effective cooperation.

Joyce et al. (2019) focused on the standard process of resilience training (specifically a mindfulness-based intervention) while employing non-traditional delivery methods. Drawing on a study involving 143 firefighters in Australia, (Joyce et al. 2019) demonstrated that psychological resilience can be strengthened through online-based training. Even when delivered entirely in an online environment, the intervention group showed a significant increase in resilience scores compared with the control group. The possibility of implementing online training on a large scale could represent a substantial advancement not only in resilience training but also in related areas of professional development.

Current empirical research in the field of security professions clearly demonstrates that psychological resilience, emotional regulation, and psychological wellbeing are key determinants of professional performance, adaptive capacity, and long-term employability among personnel exposed to high levels of occupational stress. Systematic reviews further indicate that these competencies are not merely relatively stable personality characteristics, but can be actively strengthened through structured educational and training interventions.

The need for the systematic development of resilience among members of the security forces in the Czech Republic also arises from the national institutional and legislative framework. For example, Act No. 361/2003 Coll., on the Service Relationship of Members of Security Forces (Czech Republic, 2003), emphasizes fitness for duty and the long-term sustainability of service, with psychological fitness constituting an integral component thereof.

However, international studies indicate that the system has historically prioritized physical readiness over psychological preparedness (Berlanga Silvente et al., 2026). Moreover, the psychological training of security forces is predominantly delivered retrospectively, which is not consistent with the recommendations of recent research (Moreno et al., 2024). This highlights the need to redesign the entire education and training framework, as suggested by Christopher et al. (2020). To achieve this, it will first be necessary to conduct a comprehensive analysis of the current situation.

## 3 Aim, motivation, and contribution

To date, there is a lack of comprehensive research examining resilience through an indirect approach within the context of service and professional training of the security forces in the Czech Republic. The primary aim of this article is to contribute to filling this gap in the literature and, at the same time, to outline a new framework for the education and training system in the Czech Republic that seeks to enhance emotional resilience as effectively as possible. In order to fulfill this objective, the study examines the perspectives of members of the security forces in the Czech Republic on the existing system of service and professional training, with the aim of identifying factors perceived to affect its quality, effectiveness, and practical relevance for duty performance. In addition, the research explores respondents' preferences regarding the form, content, and organization of training and seeks to formulate recommendations to support the improvement of training quality within the security forces.

To address these objectives, the study formulated a series of research questions. In light of the findings reported by McCanlies et al. (2017), the study examines the ability of the current training system to prepare individuals for demanding situations. Furthermore, in line with the findings of Singh et al. (2024), attention is given to effective teamwork (including cross-border cooperation and collaboration with external experts) as these factors play a key role in managing stressful situations. Accordingly, the study is based on a total of six research questions:

- RQ1: How do members of the security forces evaluate the current system of service and professional training in terms of its effectiveness in preparing them for psychologically demanding situations?
- RQ2: What formats of professional training do members of the security forces perceive as most beneficial for their overall preparedness?
- RQ3: What ratio of theoretical to practical components of training do respondents consider optimal?
- RQ4: How do respondents assess the relevance of teamwork, external experts, and international cooperation as components that enhance their capacity to manage high-stress operational demands?
- RQ5: What training components are perceived by respondents as supporting emotional resilience?
- RQ6: What recommendations do security personnel propose for improving the professional training system to better support their preparedness for psychologically demanding situations?

Building on the research questions, the study also formulates several hypotheses that enable the empirical examination of expected patterns in respondents' evaluations of service and professional training. These hypotheses reflect both the theoretical assumptions established in the literature and the practical considerations arising from the operational context of the security forces. Their formulation rests on the theoretical premise that a combined training approach enhances overall preparedness for service, which, in the long term, supports not only sustained employability but also the development of emotional resilience. The hypotheses also draw on the assumption that several specific factors significantly influence respondents' satisfaction with the current training system. Accordingly, the following hypotheses were established:

- H1: Members of the security forces prefer a combined model of professional training over a purely theoretical or purely practical model because it is the most beneficial to them.
- H2: Respondents with higher levels of work experience report lower satisfaction with the current training system than those with lower levels of experience.
- H3: Satisfaction with the current service training system differs significantly across respondents according to their type of service and the nature of their duty.
- H4: Respondents express an intention to improve their skills through the involvement of external experts and international partners in service training, regardless of the type of security force.

By addressing these research questions and testing the proposed hypotheses, the study provides a comprehensive overview of how members of the security forces perceive the current system of service and professional training. The findings offer empirically grounded insights into the factors that shape training quality, effectiveness, and practical relevance, as well as the preferences and expectations of personnel regarding the organization and content of professional preparation. These insights generate a valuable evidence base that can inform future developments in national (and potentially supranational) training frameworks, including areas in which systemic improvements would require legislative intervention. In this way, the study contributes both to the academic discourse on professional training in security institutions and to the practical efforts aimed at enhancing the preparedness and resilience of security forces personnel.

## 4 Data and methods

The necessary data were obtained through a questionnaire survey designed to collect feedback from experts with practical experience in security-related activities. The questionnaire was distributed online across the Czech Republic, and data collection took place in the first quarter of 2026. Anonymity was fully ensured throughout the questionnaire survey, and all respondents were informed beforehand of the study's research nature. In total, responses from 224 participants (both male and female) were included in the analysis. The dataset comprises personnel from the Customs Administration, Fire Rescue Service, Police, Office for Foreign Relations and Information, Military Police, and Prison Service.

The questionnaire consisted of two key sections. The first section included selection-type questions focusing on the personal background of the respondents. This part captured essential professional characteristics, enabling a differentiated analysis of respondents' views in relation to their professional profiles. Table 1 presents the basic characteristics of the research sample and its representativeness in relation to the specified sub-objectives of the study.

**Table 1:** Basic information about respondents.

**Characteristic**	**Frequencies**	
Type of service	Absolute	Relative (%)
Criminal police and investigation	40	17.86
Immigration police	19	8.48
Public order police	59	26.34
Traffic police	36	16.07
Other	70	31.25
Length of service	Absolute	Relative (%)
Up to 3 years	96	42.86
3–5 years	24	10.71
5–10 years	29	12.95
10–15 years	21	9.38
15–20 years	32	14.29
20 years and more	22	9.82
**Type of duty**	**Absolute**	**Relative (%)**
Direct duty	172	76.79
Indirect duty	52	23.21
Area of activity	Absolute	Relative (%)
Administrative proceedings	29	12.95
Criminal proceedings	97	43.30
Other	98	43.75
Highest level of education completed	Absolute	Relative (%)
Secondary education with school-leaving exam	131	58.48
Higher professional education	21	9.38
Bachelor's degree	49	21.88
Master's degree	16	7.14
Doctoral degree	7	3.12
Field of education	Absolute	Relative (%)
Criminalistics	46	20.54
Education/Pedagogy	5	2.23
Law	27	12.05
Security	34	15.18
Social sciences	4	1.79
Transport	12	5.36
Other	96	42.86
Position type	Absolute	Relative (%)
Non-supervisory staff	174	77.68
Second-level supervisory staff (department heads)	2	0.89
Third-level supervisory staff (section/branch heads)	24	10.71
Fourth-level supervisory staff (team and group leaders)	24	10.71

The second part of the questionnaire focused on respondents' professional experience in the field of education and training. These questions were not mandatory in the questionnaire, and therefore the number of respondents varies across the different research areas. In addition to multiple-choice items, it also included open-ended questions that allowed respondents to describe their personal experiences and viewpoints in more detail.

To analyse individual components that indirectly contribute to improvements in emotional resilience, the questionnaire included items not only addressing satisfaction with the current system of education and training, but also identifying which components respondents, based on their experience, perceived as most beneficial for enhancing their emotional resilience. The survey further examined the importance attributed by respondents to practical training (e.g., crisis intervention simulations, debriefing, and related activities) and the proportion of such training they considered optimal for better preparation for psychologically demanding situations. Attention was also paid to benefits arising from teamwork, cooperation with external entities (e.g., experts from forensic science), and the benefits of joint training activities with foreign security organizations.

Open-ended responses (used to address Research Questions RQ1–RQ6) were analyzed using qualitative content analysis. This approach was chosen because it enables a systematic, transparent, and replicable examination of textual data while remaining sensitive to the contextual nuances of respondents' statements. Qualitative content analysis is particularly suitable for studies aiming to identify patterns in perceptions, experiences, or attitudes, as it allows the researcher to move beyond surface-level descriptions and derive structured categories grounded in the data. Following established methodological recommendations (Mayring, 2022), the analysis proceeded through iterative coding, constant comparison, and the refinement of thematic categories. This procedure made it possible to identify recurring themes and respondent-driven suggestions relevant to the research objectives.

To evaluate Hypotheses H1–H4, several statistical procedures were employed. For Hypothesis H1, a proportion test was conducted using the binom.test function in the R programming language. This test was selected because it provides precise *p*-values without relying on large-sample approximations and is therefore appropriate when comparing the relative frequencies of two response categories. The two-sided exact *p*-value is defined as:

∑{k:ℙ(X=k∣p0)≤ℙ(X=x∣p0)}nkp0k(1-p0)n-k,

that is, as the probability (under the null hypothesis *p*_0_) of observing any outcome at least as unlikely as the observed value *x*. The one-sided exact *p*-value is given by:

ℙ(X≥x∣p0)=∑k=xnnkp0k(1-p0)n-k.

Hypotheses H2–H4 were assessed using the Fisher-Freeman-Halton exact test for contingency tables. This test is an extension of Fisher's exact test designed for larger *r*×*c* tables and therefore suitable for categorical variables with more than two response options. Let (*x*_*ij*_) denote the observed table with row sums *R*_*i*_, column sums *C*_*j*_, and total *N*. All possible tables with the same margins form the set:

$$T=\left\{X=\left(x_{ij}\right):\underset{j}{\sum }x_{ij}=R_{i},\underset{i}{\sum }x_{ij}=C_{j},x_{ij}\in {\mathbb{N}}_{0}\right\}.$$

Under the null hypothesis of independence, the probability of any table X∈T is given by the multivariate hypergeometric model:

$$\begin{array}{l} \mathbb{P}\left(X|H_{0}\right)=\frac{\overset{r}{\underset{i=1}{\prod }}R_{i}!\overset{c}{\underset{j=1}{\prod }}C_{j}!}{N!\overset{r}{\underset{i=1}{\prod }}\overset{c}{\underset{j=1}{\prod }}x_{ij}!}. \end{array}$$

Let $X_{\text{obs}}$ denote the observed table. The exact *p*-value is defined as:

$$\begin{array}{l} \underset{X\in T:\mathbb{P}\left(X|H_{0}\right)\leq \mathbb{P}\left(X_{\text{obs}}|H_{0}\right)}{\sum }\mathbb{P}\left(X|H_{0}\right). \end{array}$$

Since exact enumeration becomes computationally infeasible for larger tables with multiple categories, Monte Carlo sampling of contingency tables with fixed margins was applied to approximate the *p*-values. The simulations were performed in R using the fisher.test function with 2,000 replicates, ensuring a reliable estimation of the test statistics while maintaining computational efficiency. For detailed technical explanations of these analytical procedures, see Neuhäuser and Ruxton (2024).

## 5 Results

The presentation of the results in this chapter follows the order of the research questions and hypotheses outlined in Chapter 3.

### 5.1 Results related to the research questions

As part of the survey, we sought to determine whether respondents were satisfied with the current system and, where applicable, to identify the shortcomings they perceived. Drawing on responses from 162 participants (see Table 2), we were able to address Research Question RQ1 (*How do members of the security forces evaluate the current system of service and professional training in terms of its effectiveness in preparing them for psychologically demanding situations?*). Only 28 respondents (about 17%) indicated dissatisfaction with the current education system. Overall, therefore, satisfaction clearly predominates among respondents, who generally express a positive view of the current system of service and professional training.

**Table 2:** Frequency of responses—RQ1.

Respondent's response: satisfaction with the current system	Absolute	Relative (%)
I am satisfied with the current education system	134	82.72
I am not satisfied with the current education system	28	17.28

However, the analysis also revealed several weaknesses in the current system. These shortcomings appear to stem primarily from an inadequately designed educational concept. Respondents expressed a need for a system of continuing professional development that is not focused predominantly on newcomers to the profession. They emphasized that the training system should reflect current developments in the field (e.g., areas such as cybercrime and tax crime are insufficiently addressed). Many courses remain largely theoretical, and respondents would prefer a greater emphasis on practical demonstrations, procedures, and case studies applicable to real-world practice. They also recommended supplementing existing study materials with handbooks and methodological guidelines. Furthermore, attention should be given to the potential use of AI tools in everyday work tasks. Finally, respondents suggested that elements of the redesigned education system should be voluntary, allowing individuals to select certain courses based on their professional needs and interests.

Based on the responses, it can be concluded that employees would prefer a combined form of training. Table 3 presents the distribution of preferences related to Research Question RQ2 (*What formats of professional training do members of the security forces perceive as most beneficial for their overall preparedness?*). Interest in the practical aspects of training, already evident in RQ1, is further confirmed by the findings for RQ2. According to the responses summarized in Table 3, 94.44% of respondents selected either “Practical Training” or “Combined Training,” indicating a clear preference for training formats that incorporate practical components. If we were to restrict the responses from Table 3 to those respondents who expressed dissatisfaction in Table 2, all of their selections would fall exclusively into these two categories.

**Table 3:** Frequency of responses—RQ2.

Respondent's response: preferred system of professional training	Absolute	Relative (%)
Theoretical training	6	3.70
Combined system (based on solving simulated scenarios)	114	70.37
Practical training	39	24.07
Other	3	1.85

Based on these results, it can be concluded that respondents are aware of the importance of practical training, such as scenario-based exercises or simulations of stressful situations, in improving their ability to cope with demanding conditions. Such training enables personnel to learn and apply coping strategies, automate appropriate responses, and regulate their emotions more effectively in high-pressure environments. This increased level of preparedness subsequently contributes to the development of emotional resilience and supports overall mental wellbeing.

Drawing on the questionnaire responses, we are able to determine the appropriate balance between the theoretical and practical components of training, which corresponds to the scope of Research Question RQ3 (*What ratio of theoretical to practical components of training do respondents consider optimal?*). Table 4 presents the aggregated results regarding the proportion of theoretical instruction in the current training system. The most frequently selected option was a 50% theory and 50% practice ratio. On average, however, the theoretical component accounted for 60.51% of the training (leaving 38.49% for the practical component). Notably, one quarter of respondents assigned 70% or more to the theoretical component.

**Table 4:** The proportion of theoretical components in training—RQ3.

Characteristics	Share of training (%)
Lower quartile	50
Mode	50
Median	60
Average	60.51
Upper quartile	70

These responses are consistent with the findings reported under RQ1 and RQ2. The prevailing view (based on Table 3) is that the training system should combine theoretical and practical components, including the use of simulated scenarios. The current system does not fundamentally contradict this approach (as indicated by the results in Table 4), which may explain why most respondents are satisfied with the existing structure (Table 2), even though they acknowledge that there is still room for improvement.

A closer examination of the group of dissatisfied respondents within Research Question RQ1 shows that they perceive the theoretical component to be several percentage points higher (mode 80%, median 70%, average 64%). This finding suggests that the proportion of practical training should be increased slightly (by several percentage points) to better align with expectations. It can reasonably be assumed that even a modest increase of several percentage points in the practical component (if perceived by employees, even at an aggregate level) may have several positive effects. First and foremost, more intensive practical training will improve their ability to cope with difficult situations. Second, it will enhance their sense of wellbeing.

Another sub-area of the research concerns the importance of teamwork, the involvement of external experts, and international collaboration, corresponding to Research Question RQ4 (*How do respondents assess the relevance of teamwork, external experts, and international cooperation as components that enhance their capacity to manage high-stress operational demands?*). The strongest consensus was found regarding the inclusion of teamwork. A total of 138 respondents (85.19%) indicated that they would welcome group and team work as part of their professional training, whereas only 24 respondents (14.81%) stated that they would not include it.

There is, however, far less agreement on the preferred group size. Responses varied considerably, ranging from 2 to 10 participants. This likely reflects differences in respondents' professional backgrounds, as the optimal size of a group or team may depend on the topic and the specific nature of the training activity. Regardless of the size of the work teams involved, teamwork contributes to the development of social support by creating networks of mutual assistance, collaborative problem-solving, and emotional support. Such social support is a key protective factor in the development of emotional resilience, enabling employees to better manage stress, recover from adverse situations, and maintain both operational performance and mental wellbeing.

Table 5 presents the results regarding the involvement of external experts (such as specialists in forensic science) in service-related training. Almost 81% of the responses can be classified as positive—these respondents explicitly stated that this area is very important or that cooperation with external experts has been beneficial to them. Around 5% of the responses were neutral (indicating no experience or no opinion). The remaining approximately 14% of responses were negative in nature, typically suggesting that such cooperation is unnecessary.

**Table 5:** Frequency of responses–RQ4–external experts.

Respondent's response: the importance of involving external experts	Absolute	Relative (%)
It is important/It is beneficial	105	80.77
I have no opinion or experience	7	5.38
It's not important to me	18	13.85

If the involvement of external experts were structurally embedded in training programmes, it would ensure that high levels of realism, expert feedback, and specialized knowledge become a stable and predictable part of the learning process. Such institutionalization could further enhance participants' psychological preparedness and contribute to the development of emotional resilience.

Table 6 summarizes the views regarding joint training activities with foreign partners. Just under 66% of the responses can be classified as positive—these respondents expressed favorable views of such collaboration, typically stating that it is important or that they have had positive experiences. Just under 23% of the responses were classified as neutral; these were typically respondents who had no opinion, often because they had not yet had the opportunity to participate in such joint training. The remaining responses were grouped under the general category “Other” due to the highly specific nature of the comments. Among these responses, some noted that language barriers or differences in case law posed obstacles; others pointed out that opportunities for cooperation differ between basic units and other departments, that time is often used inefficiently during such collaborations, and that the overall benefit is perceived as very limited.

**Table 6:** Frequency of responses—RQ4—international collaboration.

Respondent's response: joint training exercises with international partners	Absolute	Relative (%)
It's important/I've had positive experiences	75	65.79
I have no opinion or experience	26	22.81
Other	13	11.40

While differences in nomenclature or legislative frameworks may reduce some participants' interest in this type of collaboration, they can also provide the advantage of offering a different perspective on the issue at hand. A second benefit (one that participants generally did not recognize based on their responses) is the ability to share resources and thereby reduce costs. This extends beyond access to proven know-how to include the sharing of technologies, laboratories, simulators, and similar infrastructure, which would otherwise be inaccessible, particularly to smaller countries such as the Czech Republic.

As part of our next research question, RQ5 (*What training components are perceived by respondents as supporting emotional resilience?*), we found that it is possible to identify several areas that help respondents increase their emotional resilience. Since the feedback on the open-ended questions varied widely, we categorized the responses into several categories. The most common response can be described as scenario-based stress exposure training. This primarily involves exercises conducted in real-life situations and tactical drills, which allow individuals to practice managing emotions under pressure and have a positive effect on strengthening emotional resilience. This area is closely linked to physical training (breathing control, self-control, relaxation training), which also helps improve resilience.

Another area, albeit mentioned less frequently, can be identified as debriefing, which is currently not sufficiently developed. This includes post-incident debriefs, peer discussion groups, and supervised reflection, all of which could help respondents strengthen their emotional resilience. The final pillar is associated with a well-developed and active training system. Respondents consider the existence of a lifelong training framework to be very important, one that is actively supported by supervisors. The creation of a stable and psychologically safe environment further contributes to strengthening their resilience.

Last but not least, specific suggestions for improvement were gathered directly from respondents, corresponding to Research Question RQ6 (*What recommendations do security personnel propose for improving the professional training system to better support their preparedness for psychologically demanding situations?*). These suggestions can be grouped into two categories. The first consists of proposals for system-wide changes. These systemic recommendations include, for example, increasing the proportion of practical training, offering a greater number of seminars, updating the content of courses and seminars to reflect current developments, and providing opportunities to customize selected courses according to individual professional interests. Respondents also recognized that regular knowledge verification would be appropriate (e.g., periodic re-examinations in criminal law), and that preparation for psychological tests should likewise not be underestimated.

The second area of suggestions put forward by respondents concerns specific topics that should receive greater emphasis in professional training. Respondents specifically mentioned requests for team-building activities, improved communication, greater regularity across all aspects of training, and the development of joint exercises aimed at enhancing teamwork. Additional requirements were also identified with regard to strengthening officers' physical fitness and professional development, including areas such as health education, interactions with inmates, law/case law, self-defense, and firearms training.

### 5.2 Results related to the hypotheses

As indicated in Section 5.1, there is a very strong consensus among the respondents regarding the need to combine theoretical instruction with practical demonstrations (see Table 3). In the analysis of Hypothesis H1 (*Members of the security forces prefer a combined model of professional training over a purely theoretical or purely practical model because it is the most beneficial to them*), statistical tests were conducted to assess whether the combined teaching style is preferred to the theoretical style, and whether it is likewise preferred to the practical style. Based on the very low *p*-values (< 0.001) from the tests of proportion equality, the proposed Hypothesis H1 is supported. We therefore conclude that the combined training style is indeed more favored than either the theoretical or the practical training style.

Table 7 was compiled to illustrate the data used for evaluating the second hypothesis H2 *(Respondents with higher levels of work experience report lower satisfaction with the current training system than those with lower levels of experience*). In the youngest service group (up to 3 years), there are 61 satisfied respondents (37.65%) and seven dissatisfied respondents (4.32%) with the current system. By contrast, in the group of personnel with more than 20 years of service, the number of satisfied respondents drops to only 10 individuals (6.17%), whereas the number of dissatisfied respondents decreases to just six individuals (3.70%). Although most respondents are satisfied with the current system, there appears to be a trend indicating that the proportion of satisfied individuals decreases as years of service increase. The Fisher–Freeman–Halton exact test also shows a statistically significant relationship between length of service and satisfaction with the current system, with a *p*-value of 0.031. When compared with the 5% significance level, this represents a borderline result, which may reflect a weaker underlying relationship as well as the relatively small number of respondents.

**Table 7:** Frequency of responses—H2.

**Service length**
**Satisfaction**	**Up to 3 years**	**3–5 years**	**5–10 years**	**10–15 years**	**15–20 years**	**20+ years**
Yes	61	16	13	17	17	10
No	7	5	6	1	3	6

Satisfaction was also analyzed with respect to the type of service and the nature of duty, corresponding to Hypothesis H3 (*Satisfaction with the current service training system differs significantly across respondents according to their type of service and the nature of their duty*). Only the relationship between satisfaction with the current system and whether the service is performed directly or indirectly proved to be statistically significant. The *p*-value of the Fisher–Freeman–Halton exact test was 0.016. The frequency of responses is shown in Table 8. The data indicate that dissatisfaction was particularly pronounced among those performing direct service. Individuals in the indirect service group have approximately 5.15 times higher odds of being satisfied compared to those in the direct service group.

**Table 8:** Frequency of responses—H3.

**Service type**
**Satisfaction**	**Indirect**	**Direct**
Yes	38	96
No	2	26

Within Hypothesis H4 (*Respondents express an intention to improve their skills through the involvement of external experts and international partners in service training, regardless of the type of security force*), the analysis examined whether positive attitudes toward involving external experts (such as those working in the field of forensic science) are uniform across the security forces or whether they vary according to specific areas of service. The test showed that the involvement of external experts is not related to the type of security force (Customs Administration, Fire Rescue Service, Police, Office for Foreign Relations and Information, Military Police, and Prison Service), nor to the type of service (alien police, traffic police, public order police, criminal police, and other units). The *p*-values of the Fisher-Freeman-Halton exact test were 0.770 and 0.316, respectively.

Similarly, the study examined whether general support for the involvement of international partners in training depends on the type of security force or the type of service. According to the resulting *p*-values from the Fisher–Freeman–Halton exact test (0.210 and 0.127), no relationship was found between support for the involvement of international partners and either the type of security force or the type of service.

## 6 Discussion

This study presents a practitioner-based analysis of education and training factors perceived to play an indirect, supportive role in the development of emotional resilience. Although according to expert feedback the current education and training system is not fully aligned with the findings of recent studies (e.g., Moreno et al., 2024), respondents generally reported being satisfied with the existing system in the Czech Republic. However, responses to the open-ended questions revealed several problematic areas. Based on the identified shortcomings of the current system, along with findings from other areas (see, e.g., Hoskova-Mayerova, 2016), a completely new concept for education and training has been proposed in the form of a modern framework for a systematic education and training system, designed to foster emotional resilience among members of the security forces to the greatest extent possible.

Based on the respondents' answers, preparation should not be limited to new recruits; instead, the system ought to facilitate the ongoing improvement of knowledge and skills across different areas throughout the entire duration of their service. Especially in the later stages of their professional careers, the education and training system should be at least partially voluntary, allowing individuals to choose courses that correspond to their specific needs and occupational interests. Such flexibility not only increases motivation but may also enhance adaptability by allowing personnel to continuously adjust their competencies to evolving professional demands.

Consistent with the proposal of (Lee et al. 2016), it would be advisable to incorporate a basic training module, covering both physical and psychological aspects, into the initial professional preparation of the security forces in the Czech Republic. Learning coping strategies and the fundamentals of emotional regulation at an early stage may help to prevent potential negative consequences later in working life (e.g., PTSD).

When designing both the basic and follow-up modules, it is important to ensure that tasks at each level extend beyond individuals' comfort zones while remaining manageable. Progressive increases in task difficulty facilitate the development of self-efficacy through repeated mastery experiences. By maintaining challenges within an achievable range, training reduces the risk of failure-induced disengagement while reinforcing perceived competence.

The follow-up module could be at least partially optional in order to better meet the specific needs of each individual. In the Czech civil sector, it has been demonstrated that employees' motivations (and, consequently, their needs) vary depending on their position within the organizational hierarchy (see, e.g., Lizbetinova et al., 2021). Similarly, our research has shown that satisfaction with the current system also depends on the type of service. It is therefore possible to tailor the training to meet the needs of those in both direct and indirect service roles. Such an approach may also enhance long-term employability by supporting continuous skills development and adaptability, thereby equipping personnel with competencies applicable across a range of functions, organizational contexts, and evolving professional demands.

In addition to the type of service, a statistically significant relationship was also found with length of service. This may reflect not only the number of years of accumulated experience but also generational differences. When designing the framework for an education and training system, it is essential to modernize not only the content but also the methods. For example, Generation *Z* has developed markedly different habits regarding learning methods and the technologies used, even from primary and secondary school (Hellyer, 2025). Based on the empirical findings of Joyce et al. (2019), online environments may likewise be employed – for instance, through the use of AI or training delivered via virtual reality. From the perspective of resilience, the use of modern technologies and adaptive learning environments may further enhance psychological preparedness by allowing repeated, controllable exposure to challenging situations in a safe setting.

Respondents also expressed a clear preference for training formats that emphasize practical application rather than predominantly theoretical instruction. Scenario-based and high-intensity training elements were perceived as particularly valuable, as they expose participants to demanding and dynamic conditions that resemble real-world operational contexts. Variability in training scenarios requires individuals to adjust their responses to changing and unpredictable situations, thereby fostering adaptability and flexible decision-making. At the same time, realistic simulations familiarize participants with potentially stressful conditions, which may reduce uncertainty and anticipatory anxiety prior to real-life deployment.

According to respondents, theoretical content should comprise approximately half of the overall time. The preference for an integrated preparation model combining theoretical instruction with practical training and the resolution of model situations suggests an effort to strengthen perceived competence and control in demanding working conditions. Such a balanced approach aligns with established findings in the scholarly literature identifying these factors as central to the development and maintenance of emotional resilience in high-risk professions characterized by elevated levels of stress, responsibility, and exposure to critical incidents (Rampersaud-Skorka, 2023).

Regarding theoretical knowledge, insufficient language proficiency has proven to be problematic, as it creates barriers in subsequent training involving international entities. Unlike the armed forces (where soldiers are required to pass STANAG language proficiency tests, as mandated by NATO member states) members of the police, fire service, or prison service in the Czech Republic, for example, are not subject to mandatory, universal language training of a comparable nature. Language proficiency should therefore have been incorporated even into the basic training module.

Among the recurring criticisms of the current system's content was the need to incorporate new, timely topics. The system should be more flexible and responsive to changes in the field (e.g., cybercrime or the use of AI in daily practice). Currently, there is also a lack of sufficiently developed guides or methodological manuals that staff could use both in the basic module and in subsequent education and training. Emphasis should also be placed on teamwork (typically in groups of 2–20 people). Similarly to the effective use of external experts in the civilian sphere (see, e.g., Manas and Sousek, 2010), it would be appropriate to strengthen this beneficial cooperation also in the training of the security forces. These educational approaches promote not only the development of professional skills but also social support, the sharing of experiences, and the normalization of experiences related to the performance of duties. From the perspective of emotional resilience, team training can be regarded as a preventive mechanism against isolation and burnout, strengthening collective stress management and the development of professional identity.

The proposed curriculum provides a relevant foundation for optimizing educational processes within security forces and can significantly contribute to improving the quality of training for service in a rapidly changing security environment. The framework is fully aligned with national strategic documents in the Czech Republic (such as the Concept for the Development of the Police of the Czech Republic, Ministry of the Interior of the Czech Republic, 2021). Its implementation should occur in close cooperation not only with training departments but also with psychological services. Such cooperation (including institutional support) will enable a shift from an ineffective and predominantly reactive system toward modern, prevention-oriented education and training.

It is important to note that our study does not directly measure emotional resilience as an individual psychological trait. Instead, it focuses on participants' perceptions of selected training-related factors that are commonly assumed to support its development. By examining how participants evaluate elements such as training structure, content, intensity, and experiential components, the study seeks to identify which aspects of training are perceived as most influential in fostering emotional resilience. This indirect approach allows for insight into contextual and organizational contributors to resilience development, while acknowledging that the actual level of emotional resilience cannot be empirically verified within the scope of the present research design.

All proposed changes are based on analyses derived from respondents' self-assessments and are cross-sectional in nature, which represents another limitation of this study. As the data relied on subjective evaluations, an underestimation or overestimation of certain subjective factors may therefore have occurred. In addition, the research was conducted in a single country, and the responses may thus reflect cultural specificities of that national context, in a manner comparable to findings reported by Anum et al. (2025). Nevertheless, the collected data offer valuable insights into subjectively perceived relationships between education, professional readiness, and resilience.

Future research should focus on longitudinal studies examining the impact of specific educational interventions on emotional resilience, job satisfaction, and retention among members of security forces. Long-term indicators such as staff turnover and sick leave would make it possible to assess the systemic impact of these measures. Such research could also benefit from the application of more advanced analytical techniques, as demonstrated by Bekesiene and Hoskova-Mayerova (2018), allowing for a more nuanced examination of relationships between educational inputs, psychological outcomes, and organizational indicators.

Another area not addressed by this study (but one that warrants dedicated future analysis) is the quantification of costs associated with the new curriculum framework. Changes in teaching tools, the involvement of external experts, or international cooperation may entail significant transaction costs. A systematic cost analysis should therefore be conducted, ideally resulting in a detailed overview comparable to the one presented in Holcner et al. (2014).

## 7 Conclusion

The empirical findings of this study, drawing on perception-based data collected through a questionnaire survey, indicate that the current system of service and professional training within the security forces in the Czech Republic is generally perceived by respondents as adequate. However, several patterns emerged that point to opportunities for further development. Personnel indicated that improvements in specific areas could contribute not only to enhanced professional competence but also to strengthening emotional resilience and supporting sustainable wellbeing. In particular, respondents highlighted the value of integrating external expertise, expanding international collaboration, and reinforcing the practical components of training.

These insights underline the need for training frameworks that are more experiential, better aligned with operational realities, and informed by evidence-based approaches. Incorporating these elements may help security forces personnel respond more effectively to stressful and high-pressure situations, thereby improving both individual preparedness and organizational performance. The findings therefore provide a useful basis for future revisions of training concepts and for ongoing strategic planning within the security forces.

## Data Availability

The raw data supporting the conclusions of this article will be made available by the authors, without undue reservation.
